# Knowledge, Attitude and Practice regarding the effect of yoga on periodontal health among Indian adults: A questionnaire study.

**DOI:** 10.12688/f1000research.140245.1

**Published:** 2023-10-25

**Authors:** Madhurya N. Kedlaya, Lakshmi Puzhankara, Mansi Mahendra, Vineetha K., Suraj Prasad Sinha, Anupam Singh, Shaswata Karmakar

**Affiliations:** 1 Manipal College of Dental Sciences, Department of Periodontology, Manipal Academy of Higher Education, Manipal, Karnataka, 576104, India; 2 Public Health Dentistry, Amrita School of Dentistry, Amrita Vishwa Vidyapeetham, Kochi, Kerala, 682041, India; 3 Consultant Craniofacial Orthodontist, Manipal, Karnataka, 576104, India; 4 Manipal College of Dental Sciences, Oral and Maxillofacial Surgery, Manipal Academy of Higher Education, Manipal, Karnataka, 576104, India

**Keywords:** Attitude, Knowledge, Oral health, Periodontal disease, Practice, Yoga

## Abstract

**Background:** Periodontal disease is a chronic inflammatory disease of structures surrounding the teeth. Its etiology is multifactorial. The primary etiological factor is the microbial component; the other factors are systemic, behavioral, environmental, and psychological. Conventional management includes routine periodontal therapy involving prophylactic and surgical management. In developing countries like India, complementary medicine and alternative medicines like yoga are gaining popularity for improving systemic health. Hence this pilot study was designed to assess the psychometric properties of a structured questionnaire that can assess knowledge, attitude, and practice (KAP) regarding the impact of yoga on periodontal health and systemic conditions of Indian population.

**Methods:** The KAP questionnaire was developed (Stage One) using a deductive approach, and a psychometric evaluation of the questionnaire was performed to evaluate it’s reliability and validity (Stage Two). Initial content validation and test re-test reliability were assessed using kappa statistics with binary responses. An intraclass correlation coefficient (ICC) was used to assess the questions in the practice and attitude category with categorical variables. Further assessment of psychometric properties of the questionnaire was done using item response theory. The developed questionnaire had four principal sections: demography of participants; knowledge regarding yoga and oral health; attitude towards yoga and oral health; and practice towards implementing yoga for oral health.

**Results:** The ICC for all the assessed questions was greater than 0.60 suggesting satisfactory stability. Internal consistency measured using Cronbach’s alpha for knowledge, attitude, and practice items were reported to be 0.632, 0.923, and 0.591 respectively and that of the KAP total was 0.632.

**Conclusions:** The findings of this study showed that the questionnaire had an acceptable psychometric property for measuring KAP regarding yoga and it’s role in oral and systemic health among Indian adults. The analysis of participant responses revealed that they had a medium level of knowledge regarding yoga and periodontal disease.

## Introduction

Periodontitis, the sixth most prevalent disease worldwide, has an overall prevalence of more than 11%. ^1^ Being a multifactorial infection, periodontal diseases are induced by a complex bacterial biofilm that interact with host tissues resulting in a chronic inflammatory state, breakdown of the supporting connective tissue, periodontal pocket formation, alveolar bone loss and finally, tooth loss. ^1^^–^^3^ The association between systemic diseases and periodontitis has been extensively explored. ^4^^–^^7^ Previous meta-analyses have shown that chronic periodontitis was significantly associated with overweight and obese patients as well as patients with metabolic syndrome. ^8^^–^^10^ Periodontal disease, apart from being a modifying factor of systemic health, may also impact the quality of life in the form of emotional, social and functional distress. ^11^^–^^15^ A recently published systematic review has concluded that the treatment of periodontitis improves systemic health. ^16^ Moreover, multiple risk factors have been identified as common to periodontal disease and chronic systemic diseases. ^17^ Stress is one such factor linked to both periodontal and systemic diseases.

Numerous studies have established the effectiveness of yoga in stress management. ^18^ Studies have shown that yoga decreases the level of salivary cortisol and blood glucose. ^19^^,^^20^ It also significantly decreases the heart rate and systolic and diastolic blood pressure. ^21^^,^^22^ The impact of yoga on specific health ailments, including diabetes, hypertension, cardiovascular disorder, and cancer, has been explored. These yogic techniques have been proven to improve one’s overall performance and work capacity and can act as psychophysiological stimuli to increase endogenous secretion of melatonin, which is responsible for improved mental well-being. ^23^

Exploration of the existing literature shows that the knowledge and attitude towards the practice of yoga in improving periodontal health is yet to be assessed extensively. Assessing an individual’s outlook regarding systemic and oral health maintenance using a questionnaire would help us determine their attitude toward disease prevention and control. This study was therefore designed to develop a questionnaire that can assess the knowledge, attitude and practice of Indian adults regarding yoga and its effect on systemic health and oral health with a focus on periodontal health.

## Methods

### Ethical approval and consent

Ethical approval was obtained from the Institutional Review Board of Kasturba Medical College (KMC) and the Kasturba Hospital Institutional Ethics Committee (IEC-841/2020, obtained on 6th February 2021) which is the Institutional Ethics Committee of Manipal Academy of Higher Education. Informed consent was obtained from all participants via electronic mode using the Google form. The first page of the Google form gave information about the study and obtained consent from the participants following which they were able to move to the subsequent pages containing the questions. The participants were completely anonymized and delinked.

### Research design and study population

This questionnaire study was conducted between February 2021 and May 2022 among Indian adults. Ethical clearance was obtained for the study from the institutional ethical committee. The study was a pilot study designed to determine the psychometric properties of the KAP questionnaire. The content validation of the questionnaire was performed by five exponents in the field of yoga and health care. Following content validation, the reliability of the questionnaire was tested. The questionnaire was subsequently distributed as a Google form to collect data for assessment of additional psychometric properties to develop a validated and reliable questionnaire.

### Sample size and sampling method

Sample size was determined based on the rule of thumb that states that five respondents are required for each item as suggested by Kline R.B. ^24^ Convenience and snowball sampling methods were adopted to include patients reporting to the Department of Periodontology, Manipal College of Dental Sciences, for dental health problems. The contact details were obtained from participants’ acquaintances as a part of snowball sampling. A web-based questionnaire was sent (Online survey by Google Forms) to the prospective participants along with a letter of invitation to participate. Indian adults who were willing to participate in this study and had knowledge of the English language were included, while those not willing to participate were excluded. Anticipating a 50% lack of response, the questionnaire was sent to twice the required participants.

### Questionnaire development

The Knowledge, Attitude, and Practice questionnaire was developed (Stage One) using a deductive approach and a psychometric evaluation of the questionnaire was performed to evaluate reliability and validity (Stage Two).

*Stage One: Item and domain development*

Using a deductive approach, questionnaire items were developed from the literature on yoga in managing periodontal disease and its influence on systemic diseases. Eight articles were referenced and conceptual definitions of knowledge, attitude, and practice regarding yoga and periodontal disease were derived. ^23^^,^^25^^–^^31^ Knowledge was defined as the awareness of what yoga is and its role in managing systemic diseases, stress, and oral diseases. The definition of attitude was given as the manner in which an Indian adult would think and behave toward the possibility of the application of yoga in addressing systemic and oral diseases. Practice was defined as the scheme followed by Indian adults who demonstrate an understanding of the use of yoga in dealing with systemic and oral diseases.

An initial questionnaire with appropriate items was developed based on related literature in English language to fit the categories and the total number of items in the questionnaire was 36. The initial KAP questionnaire was reviewed by an expert panel comprising experts on yoga and periodontal health to validate the content. The test-re-test reliability of the questionnaire was calculated by administering the scale to 30 participants twice within one month. There were 27 items and 4 domains in the final questionnaire; (1) demography of participants; (2) knowledge regarding yoga and oral health; (3) attitude towards yoga and oral health; and (4) practice towards implementing yoga for oral health. Periodontal disease and health was the primary area of focus in the oral health aspect of the questionnaire can be found in the Extended data.

*Stage Two: Psychometric evaluation*

A sample size of 135 was determined based on the rule of thumb given by Kline R.B. ^24^ and repeated in the study by Selvaraj *et al.* ^32^ The questionnaire was administered online and informed consent was obtained via electronic mode. The descriptive analysis and assessment of Item response theory ^33^ were performed using the JMETRIK software (version 4.0.0, Charlottesville, Virginia, USA).

*Item Response Theory (IRT)*

A sample size of 135 participants was considered acceptable for IRT analysis of the knowledge domain. For the knowledge domain, one-parameter logistic item response theory (1-PL IRT) analysis was carried out with the response as either right or wrong as a dichotomous output. The analysis was performed in JMETRIK using the RASCH function of the ltm package. A range of difficulty (−4 to +4) was considered as the cut-off value for the psychometric Property evaluation of the domain. ^34^

*Internal consistency reliability*

The internal consistency (IC) of the items was calculated using the coefficient of Cronbach’s alpha and correlation between items.

## Results

### Characteristics of the study population

The majority of the participants were in the age group of 31-40 years (39.7%) followed by 23-30 years (25.6%) of age. 50.8% of the participants were female and 56.3% had completed their post-graduate education. Most of the participants in the study had no history of systemic illnesses (80.9%). Diabetes had the highest percentage of occurrence (5.5%) amongst the remaining 19.1%. Oral health care was found to be an ancillary consideration as the question on frequency of visits to the dentist for consultation regarding oral or gum health was met with the answer from the majority (63.8%) that they visited the dentist only in the presence of dental problems, 12.6% said that they visited the dentist once in a year and 11.1% had never visited a dentist. 88.9% had no behavioral risk factors such as alcohol consumption, tobacco usage, and a sedentary lifestyle. Alcohol consumption, including regular consumption (5%), occasional drinking (1%), and alcohol consumption along with smoking (2.5%), was the leading behavioral risk factor.

### Questionnaire development

*Total items developed using deductive approach and content validation (Stage One)*

After consulting relevant literature, an initial questionnaire with 36 items was developed using the deductive approach. Five experts performed content validation of each scale to ensure content relevance, representativeness, and technical quality. The Scale level Content Validity Index/Average (S-CVI/Ave) for the questionnaire was 0.88. The Scale level Content Validity Index/Universal Agreement (S-CVI/UA) was 0.414. The Free Marginal Kappa was 0.53, indicative of moderate agreement, Fixed Marginal Kappa was -0.13, and the percentage agreement was 76.55%. Item reduction was performed to 30 after eliminating six questions after Content validation. The Content Validation Index (CVI) was found to be satisfactory.

*Test-re-test reliability*

Item scoring was finalized and the questionnaire was subjected to test-re-test reliability assessment. Unweighted Kappa Coefficient was used to assess the reliability of the items with binary responses. One question in the Knowledge category had poor agreement (K2: -0.036) and two had slight agreement (K3: 0.151, K4: 0.081). All the other questionnaire questions had a moderate, substantial, or near-perfect agreement.

Intraclass correlation coefficient (ICC) was used for assessing the questions in the practice and attitude category with categorical variables. The interpretation of the ICC was given as acceptable if values were above 0.40, but probably improvable; ICCs above 0.60 or greater indicated satisfactory stability; ICCs greater than 0.80 specified excellent stability. The ICC for all the assessed questions was greater than 0.60 suggesting satisfactory stability.

Three questions from the knowledge category were thus eliminated after the test- re-test reliability assessment.

*Psychometric evaluation of the questionnaire (Stage Two)*

The 27-item questionnaires were administered to 300 participants through the online platform to compensate for the expected non-response. From this, 199 responses were obtained, and all the responses were evaluated to assess the psychometric properties of the questionnaire. The one-parameter model for the knowledge domain of the respondents revealed an acceptable range of difficulty for all the questions with the values ranging from 1.01 to 2.16 ( Table 1).

**Table 1.** Item Response Theory (Rasch Model).

Item	Difficulty (SE)	P value
K1	1.21 (0.24)	0.4869
K2	1.01 (0.27)	0.1577
K3	1.27 (0.20)	0.5931
K4	1.68 (0.28)	0.5435
K5	1.27 (0.21)	0.8248
K6	2.16 (0.44)	0.5864
K7	1.21 (0.20)	0.9334
K8	1.28 (0.26)	0.4243

Internal consistency measured using Cronbach’s alpha for knowledge, attitude and practice items were reported to be 0.632, 0.923, and 0.591, respectively and that of KAP total was 0.632 ( Table 2a). Guttman Split-Half Coefficient was satisfactory for knowledge (0.600) and attitude (0.781) ( Table 2b).

**Table 2a.** Reliability.

Item	Cronbach’s Alpha
K Total	**0.632**
A Total	**0.923**
P Total	**0.591**
KAP Total	**0.632**

**Table 2b.** Guttman Split-Half Coefficient was satisfactory for knowledge and attitude.

	Guttman Split-Half Coefficient
Knowledge	0.600
Attitude	0.781

### Association of KAP with demographic variables

An independent t-test was performed to assess the association between demographic variables such as age, gender, education, and KAP regarding the application of yoga in oral health. A significant association was seen between the level of education and KAP of yoga and oral health ( Table 3).

**Table 3.** Association of KAP with demographic variables.

Variable	Mean	SD	P value
**Age**			
Below 50	**37.0276**	**7.9529**	**0.182**
Above 50	**37.7917**	**6.3913**	
**Gender**			
Male	**37.9286**	**8.2976**	**0.366**
Female	**36.4554**	**6.7372**	
**Education**			
Under graduation	**39.1467**	**8.6285**	0.040 *
Post graduation	**35.9919**	**6.5957**	

Independent t-test; p value >0.05.

* P value is significant.

### Standardization of scores

For the Indian population, the 27 item questionnaire scores were standardized. For the questionnaire, scores below 33 indicated low KAP, 34-40 indicated medium KAP and scores greater than 41 indicated good KAP of yoga for overall health, including oral health with a focus on periodontal health. The current study’s total KAP was 37.1859, indicating a medium KAP ( Table 4).

**Table 4.** KAP of Yoga for overall health including oral health.

	Mean	Standard deviation
**Knowledge**	11.3970	3.10883
**Attitude**	14.9497	4.85731
**Practice**	10.8391	4.0169
**Total**	37.1859	7.56124

### Frequency of responses in Knowledge, Attitude and Practice questions

82.4% of the participants felt that oral health is a mirror of general health and 92% were aware of the general symptoms of periodontal disease, such as bleeding gums, loose teeth, and receding gums. Although 53.3% had known that stress at work or in life could contribute to oral diseases, 35.7% were not aware of the significance of stress on oral health. 81.9% of the participants agreed that physical activity/lifestyle changes would help control chronic diseases, while only 60.8% felt the same about the influence of physical activity/lifestyle changes on gum diseases. 98% of the participants knew that yoga is a group of physical, mental, and spiritual practices of disciplines that originated in ancient India and 86.9% were knowledgeable about specific ‘asanas’ to reduce stress, blood sugar level, blood pressure and inflammation. Only 57.3% recognized that there are scientific studies that have proven the benefits of yoga on systemic health and oral/gum health.

The majority of the participants agreed that yoga is necessary for a healthy life (51.8%-Strongly agree, 32.7% agree), that yoga practice can help in managing lifestyle diseases like obesity, hypertension, cancer (50.3%-Strongly agree, 39.7% agree) and that yoga practice can improve memory and concentration (58%-Strongly agree, 32.2% agree). The participants also felt that yoga could help manage mental ill health like depression, stress (57.3%-Strongly agree, 36.2% agree) and facilitate healing, relaxation and reduction in pain (47.7%-Strongly agree, 39.7% agree). The majority of the participants were of the attitude that gum disease/periodontal disease is caused due to stress, defective immunity of the body in addition to poor oral hygiene (25.6%-Strongly agree, 44.7% agree) and that the effect of yoga on periodontal and oral health may be due to its effect on immunity and inflammation (Strongly agree-20.6%, Agree-38.2%). However, they were sceptical about the role of yoga in managing gum diseases/periodontal diseases (Neutral-46.7%, Disagree-5.5%, Strongly disagree-0.5%) and its effect on salivary flow and dry mouth, which often result in periodontal diseases (Neutral-47.2%, Disagree-3.5%, Strongly disagree-0.5%).

Amongst the practice questions, 42.7% of the respondents practiced yoga, with 52.9% practicing yoga regularly. Lack of time was the most common reason for the non-practice of yoga (29.8%). 54.1% practice yoga for less than 30 minutes and 43.2% are focused on the various asanas and 27% perform meditation. Many of the participants (97.6%) practice yoga for the improvement of general health while 27.1% focus on yoga for specific ailments. 97.6% find yoga beneficial in improving general health or specific ailment. 88.2% of the participants have not considered the practice of yoga for the health of their gums which is primarily due to a lack of awareness regarding the effects of yoga on oral health (68%), but 66.7% have noticed that the health of the gums improve with the practice of yoga. 70.9% of the participants are very willing to recommend yoga for the improvement of general health/specific ailment to others and only 4.5% are not willing. However, only 35.7% are willing to recommend yoga to improve the health of gums to others and 15.1% are unwilling to do so.

## Discussion

Yoga is a form of Complementary and Alternative Medicine (CAM) practiced in India. It is derived from the Sanskrit word ‘yuj’. ^35^ It means to join and to direct and concentrate one’s attention. The increase in modernization, with an increase in sedentary lifestyle, has led to a rise in chronic systemic diseases. Hence, lifestyle modification is the need of the hour and yoga has been gaining popularity worldwide for achieving mental and physical health.

Yoga can impact the overall well-being of an individual through several mechanisms and one of its significant influences is the effect on stress. Studies have demonstrated that the antioxidant levels of the body can be maintained or improved with the regular practice of yoga which will help regulate the antioxidant defence system under stressful conditions. ^36^ Yogic breathing exercises can also reduce the levels of free radicals. ^37^ These effects may prove helpful to maintain systemic health with an additional beneficial influence on periodontal health. However, the studies on the knowledge regarding the possible impact of yoga on periodontal health are limited. Hence, this study was designed to overcome the paucity in the literature pertaining to this topic. Apart from this, the knowledge and attitude of the populace regarding aspects of general health and oral health have also been explored in this study.

This study has facilitated the development and validation of a questionnaire for assessing Knowledge, Attitude, and Practice regarding the effect of yoga on overall health and oral health, including periodontal health among Indian adults. To our knowledge, this is the first study to develop a validated questionnaire for this purpose. After item reduction following validation by an expert panel, 30 questions were included for test re-test reliability assessment. Three questions from knowledge were eliminated after assessing the test re-test reliability. Test-retest reliability is the degree to which test scores remain unchanged when measuring a stable individual characteristic on different occasions. ^38^ Evaluation of the psychometric properties of the questionnaire was done by item response theory using the Rasch model and an acceptable range of difficulty was seen for all the questions. ^39^ Internal consistency assesses the consistency of results across items within a test and in this questionnaire, this property, as measured using Cronbach’s alpha, ^40^ showed a total KAP of 0.632 and all factor loadings were more than 0.3 showing a close association among factors and items. Guttman Split-Half Coefficient is considered a measure of the internal reliability of a test. This test confirms how consistently the items perform within a test. ^41^ It was satisfactory for knowledge and attitude components of this questionnaire.

When assessing the demographic details, a significant association was seen between the level of education and KAP of yoga for overall health, including oral health. This points towards education as one of the factors that can influence the practice of yoga for overall health and oral health. This may be attributed to the fact that there is an increase in the interest towards incorporation of yoga in the school curriculum and hence there is more awareness regarding the benefits of yoga on health.

Majority of the participants in this study were aware that oral health is a critical aspect of general health and could identify the initial symptoms of periodontal diseases. In a study on children by Nagarajappa *et al.*, it was observed that a large proportion of children were not conscious of health risks originating from poor oral health. ^42^ In contrast, another study showed that students had adequate level of knowledge on causes, prevention, and signs of dental caries and periodontal diseases. ^43^ Findings from another study in adults showed that poor knowledge regarding oral health and its significance was associated with participants age, education, ethnicity, income and reading ability. ^44^

In this study, over 50% of the participants identified stress as a potential risk factor for periodontal disease. The role of physical activity or lifestyle change in improving systemic health was asserted by majority of the participants in this study although they were doubtful about the effect of the same on oral health. Stress and physical inactivity have been recognized as few of the multiple risk factors for systemic and oral diseases. Research has indicated a statistically significant relationship between Body Mass Index levels, oral hygiene, eating habits and physical activity. ^45^ However, the awareness regarding the factors is sparse. The findings from our study are corroborated with evidence from literature that have demonstrated that patients are often aware of the role of plaque bacteria on periodontal disease, however, their knowledge of the role of stress or obesity on periodontal disease is limited. ^46^

Only 42.7 % population practiced yoga and it was observed that the most common cause for the nonpractice of yoga was lack of time. This is similar to the result obtained from questionnaire surveys conducted by Sharma G *et al.*, Hegde VS *et al.* ^47^^,^^48^ Various forms of yoga were performed by the study participants and included asanas, meditation, and pranayama focusing on general health. Most of the participants were of the opinion that yoga can help improve the overall health, both mental and physical, of an individual and they showed a positive response for willingness to recommend yoga to the general population to improve general health. The participants who performed yoga did not focus specifically on oral disease or gum disease as they lacked awareness regarding the potential effect of yoga on oral or periodontal health. A few of the participants did find an improvement in oral health as a result of performing yoga. At the same time, only a minority expressed willingness to recommend yoga for oral health or the health of gums as most of them were unconvinced of the probable role of yoga on periodontal health. A literature search has shown articles that have identified the beneficial response of the human body to the practice of yoga, ^49^^,^^50^ but the influence of yoga specifically on oral health, is less explored.

In the present study, the KAP regarding the effect of yoga on oral health is at a medium level. This may be attributed to the fact that there are a few studies that have identified the favourable effects of yoga on oral health and specifically periodontal health, ^28^ and this information is not clearly understood by the general populace. Hence, increasing awareness regarding the potential advantageous effects of yoga on oral health can pave the way toward a healthy oral cavity in a healthy body. However, the study has limitations with respect to the sampling method involved and therefore, well-defined clinical trials may be used to get an unambiguous data on the potential effects of yoga on periodontal health.

## Conclusion

There are questionnaires that evaluate oral health knowledge, attitude, and practice, but the current questionnaire content will be an ideal tool to assess the KAP regarding yoga and systemic health and the effect of yoga on periodontal health. It can be used for future studies as it has acceptable validity and reliability outcomes.

The knowledge, attitude and practice regarding yoga and its effect on periodontal health has to be investigated further so that it can be understood clearly, and steps may be implemented to facilitate integrating professional and personal oral health care with physical activity including yoga for maintenance of oral health and overall health.

## Data Availability

Figshare: Knowledge, Attitude and Practice regarding the effect of yoga on periodontal health among Indian adults: A questionnaire study. https://doi.org/10.6084/m9.figshare.24003657.v2. ^51^ This project contains the following underlying data:
- Data coded.xlsx - Yoga KAP results.docx (aggregated data) Figshare: Knowledge, Attitude and Practice regarding the effect of yoga on periodontal health among Indian adults: A questionnaire study. https://doi.org/10.6084/m9.figshare.24003657.v2. ^51^ This project contains the following extended data:
- Content validation.docx - Reliability- Test-Retest.docx - Questionnaire Knowledge, Attitude and Practice regarding periodontal and systemic diseases.pdf Figshare: STROBE checklist for “Knowledge, Attitude and Practice regarding the effect of yoga on periodontal health among Indian adults: A questionnaire study”. https://doi.org/10.6084/m9.figshare.24003657.v2. ^51^
