# Supplementary material for: Development and validation of a structured questionnaire to assess the Knowledge, Attitude and Practice (KAP) regarding the effect of yoga on periodontal health among Indian adults
Source: F1000Res. 2024 May 17;12:1404. Originally published 2023 Oct 25. [Version 2] doi: 10.12688/f1000research.140245.2 (PMC11157492; doi:10.12688/f1000research.140245.2)
Supplement: Peer review history

# Reviewer report: Reviewer response for version 2

**DOI:** 10.5256/f1000research.166342.r279735

**Published:** 2024-06-05

**Authors:** Baiju R M

**Affiliations:** 1 Government Dental College, Kottayam, Kerala, India

Revision has been satisfactorily done.

Is the work clearly and accurately presented and does it cite the current literature?

Partly

If applicable, is the statistical analysis and its interpretation appropriate?

Yes

Are all the source data underlying the results available to ensure full reproducibility?

Partly

Is the study design appropriate and is the work technically sound?

Yes

Are the conclusions drawn adequately supported by the results?

Partly

Are sufficient details of methods and analysis provided to allow replication by others?

Partly

Reviewer Expertise:

Scale development and QoL research, Implant dentistry and periodontal regeneration

I confirm that I have read this submission and believe that I have an appropriate level of expertise to confirm that it is of an acceptable scientific standard.

# Reviewer report: Reviewer response for version 1

**DOI:** 10.5256/f1000research.153580.r218411

**Published:** 2024-05-14

**Authors:** Rajeshwari H R

**Affiliations:** 1 University of Toronto, Toronto, Ontario, Canada

Knowledge, Attitude and Practice regarding the effect of yoga on periodontal health among Indian adults: A questionnaire study.

 I hereby congratulate the authors on exploring an interesting topic that may have close correlation with oral health particularly periodontal status. I am pleased to approve the  manuscript. There are primarily few minor comments and one major comment authors need to address. Please see below.

 Major Comment: Authors need to highlight at the end of the introduction how the data from this current study would affect the diagnosis or management of periodontal diseases, if it does. This would help the readers and clinicians to understand the need of the study and conduct clinical studies in this direction if it will benefit the periodontal management in a holistic way.

 Minor comments:

 Did authors find any differences in the results and socioeconomic status of the participants?

 Can authors explain how was the 50% lack of response to questionnaire anticipated? Please add if there are any reference or if there was any pilot study conducted.

Is the work clearly and accurately presented and does it cite the current literature?

Yes

If applicable, is the statistical analysis and its interpretation appropriate?

Yes

Are all the source data underlying the results available to ensure full reproducibility?

Yes

Is the study design appropriate and is the work technically sound?

Yes

Are the conclusions drawn adequately supported by the results?

Yes

Are sufficient details of methods and analysis provided to allow replication by others?

Yes

Reviewer Expertise:

Perioontology

I confirm that I have read this submission and believe that I have an appropriate level of expertise to confirm that it is of an acceptable scientific standard.

# Reviewer report: Reviewer response for version 1

**DOI:** 10.5256/f1000research.153580.r218412

**Published:** 2024-02-29

**Authors:** Baiju R M

**Affiliations:** 1 Government Dental College, Kottayam, Kerala, India

1. Further analysis of existing literature regarding the KAP of Yoga or similar physical activities and their influence on oral and periodontal health may be discussed in the introduction and discussion.
2. Methodology of item generation and item reduction may be explained further. The qualitative approaches employed may be further elaborated. The final questionnaire is in accessible.
3. Conclusions does not reflect exactly the objectives and observations.
4. The title is not reflecting the tool development that was the primary objective of the study.

Is the work clearly and accurately presented and does it cite the current literature?

Partly

If applicable, is the statistical analysis and its interpretation appropriate?

Yes

Are all the source data underlying the results available to ensure full reproducibility?

Partly

Is the study design appropriate and is the work technically sound?

Yes

Are the conclusions drawn adequately supported by the results?

Partly

Are sufficient details of methods and analysis provided to allow replication by others?

Partly

Reviewer Expertise:

Scale development and QoL research, Implant dentistry and periodontal regeneration

I confirm that I have read this submission and believe that I have an appropriate level of expertise to confirm that it is of an acceptable scientific standard, however I have significant reservations, as outlined above.

# Response

**Published:** 2024-03-01

**Authors:** LAKSHMI PUZHANKARA

**Affiliations:** MCODS, Periodontology, Manipal Academy of Higher Education, Manipal, Karnataka, India

Dear Sir,

 Thank you very much for your comments. We shall incorporate the suggestions you have given and make the changes in the article.
